# An Explorative Biomarker Study for Vaccine Responsiveness after a Primary Meningococcal Vaccination in Middle-Aged Adults

**DOI:** 10.3389/fimmu.2017.01962

**Published:** 2018-01-11

**Authors:** Marieke van der Heiden, Guy A. M. Berbers, Susana Fuentes, Menno C. van Zelm, Annemieke M. H. Boots, Anne-Marie Buisman

**Affiliations:** ^1^Centre for Infectious Disease Control (Cib), National Institute for Public Health and the Environment (RIVM), Bilthoven, Netherlands; ^2^Department of Rheumatology and Clinical Immunology, University of Groningen, University Medical Centre Groningen, Groningen, Netherlands; ^3^Department of Immunology, Erasmus MC, Rotterdam, Netherlands; ^4^Department of Immunology and Pathology, Monash University and Alfred Hospital, Melbourne, VIC, Australia

**Keywords:** biomarkers, vaccine responsiveness, middle-aged adults, regulatory T cells, CD4 T cells, primary vaccination

## Abstract

**Introduction:**

Prevention of infectious diseases in the elderly is essential to establish healthy aging. Yet, immunological aging impairs successful vaccination of the elderly. Predictive biomarkers for vaccine responsiveness in middle-aged adults may help to identify responders and non-responders before reaching old age. Therefore, we aimed to determine biomarkers associated with low and high responsiveness toward a primary vaccination in middle-aged adults, for which a tetravalent meningococcal vaccine was used as a model.

**Methods:**

Middle-aged adults (50–65 years of age, *N* = 100), receiving a tetravalent meningococcal vaccination, were divided into low and high responders using the functional antibody titers at 28 days postvaccination. A total of 48 parameters, including absolute numbers of immune cells and serum levels of cytokines and biochemical markers, were determined prevaccination in all participants. Heat maps and multivariate redundancy analysis (RDA) were used to reveal immune phenotype characteristics and associations of the low and high responders.

**Results:**

Several significant differences in prevaccination immune markers were observed between the low and high vaccine responders. Moreover, RDA analysis revealed a significant association between the prevaccination immune phenotype and vaccine responsiveness. In particular, our analysis pointed at high numbers of CD4 T cells, especially naïve CD4 and regulatory T subsets, to be associated with low vaccine responsiveness. In addition, low responders showed lower prevaccination IL-1Ra levels than high responders.

**Conclusion:**

This explorative biomarker study shows associations between the prevaccination immune phenotype and vaccine responsiveness after a primary meningococcal vaccination in middle-aged adults. Consequently, these results provide a basis for predictive biomarker discovery for vaccine responsiveness that will require validation in larger cohort studies.

## Introduction

Prevention of infectious diseases in the elderly is essential to establish healthy aging in the rapidly growing aging population. Yet, immunological aging impairs successful vaccination in the elderly (1–3). Timely vaccination of middle-aged adults may be an alternative option to strengthen the memory immunity before reaching old age. Previously, we showed that a primary meningococcal vaccination, containing antigens toward which no or very low prevaccination immunity exists, was highly immunogenic in middle-aged adults (4). Moreover, we described the induction of T cell responses by the tetanus toxoid (TT) carrier protein that are in favor of efficient T cell help (5). Current research focusses on the identification of immune markers in older individuals to be able to predict the vaccine responders and non-responders (6, 7). At present, the discovery of these predictive immune markers at advanced age is challenging and results are not unambiguous.

Potential biomarkers for vaccine responsiveness may relate to shifts in the immune phenotype from naïve to more memory cells during aging. This phenomenon occurs especially in the T cell compartment and is caused by thymus involution (8–13). Accordingly, the responsiveness to a yellow fever vaccine was found positively associated with the numbers of circulating naïve CD4 T cells that had recently left the thymus (14). Infection with persistent viruses, such as cytomegalovirus (CMV), enhances the numbers of late-differentiated T cells and consequently may accelerate immunological aging (15, 16). High numbers of these late-differentiated T cells were negatively associated with influenza and varicella zoster (VZV) vaccine responses (17, 18). In addition, increased numbers of regulatory T (Treg) cells are observed at old age (19, 20) which may underlie the lower responsiveness to the influenza and VZV vaccinations (18, 21).

Age-associated changes in the B cell compartment have also been reported and include a decrease in naïve B cells and a subsequent increase in late-differentiated and exhausted B cells, as well as B cells with inflammatory characteristics (22–24). Several vaccination studies described a positive correlation between the frequencies of prevaccination Ig switched memory B cells and the responsiveness to influenza and hepatitis B vaccines (17, 23, 25–27), whereas late/exhausted (CD27–IgD−) memory B cells were negatively correlated with the response to the influenza vaccine (23). Moreover, B cell expression levels of activation-induced cytidine deaminase (AID) and TFN-α after *in vitro* stimulation were found predictive for humoral responses after influenza vaccination (23, 26, 28–30).

In addition, several innate immune functions, gene signatures, or miRNA expressions were associated with influenza vaccine responsiveness (25, 31, 32). Moreover, the age-associated increase in inflammatory mediators, also known as “inflammageing” (33–36), as well as modified expression of biochemical markers, such as dehydroepiandosterone sulfate (DHEAs) (37) and vitamin D (38), might affect the immune function at advancing age (39). Also, a range of vaccination responses, for example, to diphtheria, tetanus, and influenza, are substantially influenced by vaccine-specific prevaccination immunity (27, 31).

The studies mentioned provide some promising predictive biomarkers that require validation in other cohort studies. In the present study, we aimed to explore differences in the prevaccination immune phenotype between low and high vaccine responders toward a primary immune response upon a meningococcal vaccination in middle-aged adults.

## Materials and Methods

### Study Design

**Figure 1:**
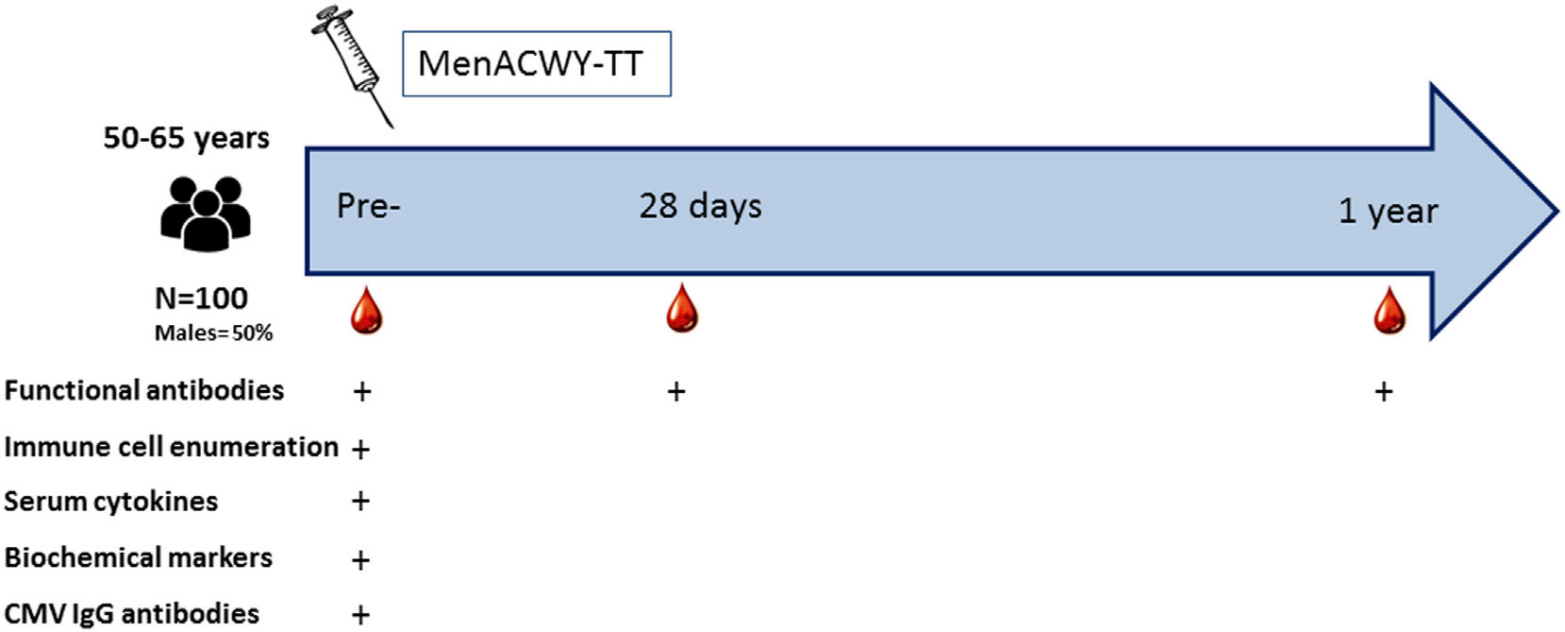
Study outline.

Data from 100 middle-aged (50–65 years of age, 50% males) adults who received the tetravalent meningococcal vaccine conjugated to TT were used in this explorative biomarker study. These participants were included in a larger cohort study, of which exclusion criteria and study procedures are described elsewhere (4). In short, prevaccination blood samples were drawn from all participants as well as 28 days, and 1-year postvaccination blood samples. Serum samples were collected at the different time points using serum clotting tubes (BD Biosciences) and were immediately kept cold and stored within 4 h in aliquots at −20 and −80°C before further use. Blood samples were collected in tubes containing lithium heparin (BD Biosciences) for detailed cellular immune phenotyping prior to vaccination. Subsequently, different immune parameters, i.e., absolute immune cell counts, serum cytokines, CMV-specific antibodies, and biochemical markers were measured in the prevaccination blood samples of these participants. Meningococcal-specific functional antibody titers were measured in the prevaccination, as well as 28 days and 1-year postvaccination samples. A schematic overview of the study outline is depicted in Figure 1. In addition, all participants filled in a short health questionnaire.

### Participant Selection

Functional antibody titers for the three different meningococcal groups (Meningococci C, W, and Y) were measured with the serum bactericidal antibody assay in 100 middle-aged adults, as previously described (4, 40, 41). Meningococci-A-specific analysis was left out, due to interference of cross-reactive antibodies in the antibody assays. A functional antibody titer of 8 was considered to be protective, whereas a functional antibody titer of 128 was applied as a more conservative long-term correlate of protection (4, 40).

The quartiles of the functional antibody titers 28 days postvaccination were calculated. Participants with a functional antibody titer matching the corresponding titer of the first quartile or below were considered low responders, whereas those matching the titer of the third quartile or above were considered high responders. Since part of the participants showed antibody titers equal to the cutoff value, the lowest and highest quartile do not include 25% of the participants. In total, 25, 46, and 40 low responders and 27, 35, and 34 high responders were defined for MenC, MenW, and MenY, respectively.

### Flow Cytometric Analysis

At the prevaccination time point, the absolute numbers of a broad range of immune cell subsets were determined as described previously (42–44). In brief, the absolute numbers of lymphocytes, T cells, B cells, NK cells, monocytes, and granulocytes were measured in fresh whole blood samples (within 18 h after collection) using TruCOUNT tubes. Gating strategies as well as a detailed description of the antibodies used were as published previously (42). Example gating strategies are shown in Figure S4 in Supplementary Material. An overview of the phenotype definitions of the different cellular subsets measured is depicted in Table S1 in Supplementary Material. These absolute cell numbers were also used to calculate the ratios between the (memory) Treg cells and the CD4^+^CD45RO^+^ effector memory T (TemRO) cells as well as between the CD4 naïve or CD4^+^CD45RA^+^CD25^dim^ cells and the CD4 memory cells. The CD4 memory cells were defined as the sum of the CD4 central memory (CM), CD4 TemRO, and CD4^+^CD45RA^+^ effector memory T (TemRA) cells.

### Serum Cytokines

A set of inflammatory (TNF-α, MCP-1, soluble CD40L, and IL-6) and one anti-inflammatory (IL1 receptor antagonist (IL-1Ra)) serum cytokines were measured in serum samples that were kept cool right after harvest and stored at −80°C within 4 h and prevented from freeze-thaw cycles. Multiplex immunoassays (MIAs) were used to measure the serum cytokine levels as described previously (45, 46). Since serum levels of IL-6 were below detection limit, IL-6 was left out of the analysis.

### Serum Biochemical Parameters

Serum levels of C-reactive protein (CRP; mg/L), Rheumatoid factor (RF; IU/mL), reactive oxygen metabolites (ROM; IU/L), and total thiol (TTT; μmol/L) were measured with a clinical auto-analyzer (Dx5, Beckman-Coulter). Dehydroepiandrosterone sulfate (DHEAs; μmol/L) and 25-hydroxyvitamin D (VitD; nmol/L) were measured using the immuno-analyzer Acces-2 from Beckman Counter.

### Statistical Analyses

The functional antibody titers were compared between the high and low responders with the Mann–Whitney *U*-test. The geometric means with the 95% confidence intervals (CIs) are indicated in the graphs. The chi-square test was used to determine significant differences in patient characteristics between the high and low vaccine responders.

The different immune markers were compared between the high and low responders using the Mann–Whitney *U*-test. Furthermore, the group-specific geometric mean values of the different immune markers were normalized to *z*-scores using the geometric means and standard deviation of the total group of 100 participants. The normalized *z*-scores were displayed on a color scale in the heat maps, ranging from red (below the geometric mean of the total group) to blue (above the geometric mean of the total group). The color darkness is representative of the deviation from the total group geometric mean. For these analyses, SPSS V22.0 and Graphpad Prism V7 were used.

Multivariate redundancy analysis (RDA) was used to asses associations between vaccine responsiveness and the prevaccination immune phenotype. The absolute numbers of immune cells as well as the levels of serum cytokines and biochemical markers were imported in the analysis as biological variables, whereas vaccine responsiveness, age, sex, and CMV were included as explanatory variables. Significance of the explanatory variables was assessed by Monte Carlo permutation testing (MCPT). The *p*-values as well as the false discovery rates (FDRs) are given. Biological variables with the highest variation explained by the explanatory variables are depicted in the plots (FitE > 15). Canoco5 software for Windows (47) was used to perform this analysis. A value of *p* of < 0.05 was considered statistically significant.

## Results

### Participant Characteristics

**Figure 2:**
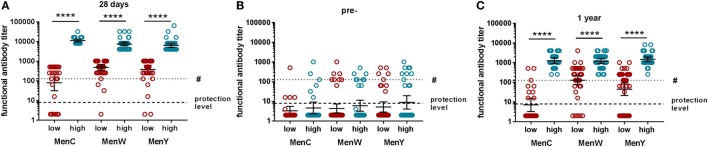
The meningococcal group-specific functional antibody titers of the low (red) and high (blue) vaccine responders. The functional antibody titers 28 days **(A)** postvaccination, prevaccination **(B)** and 1-year postvaccination **(C)** of the low (red) and high (blue) vaccine responders. The protection level is indicated by the lowest dotted line. A more conservative long-term protection level is indicated by the # in the figures. The functional antibody titers were compared between the low and high responders using the Mann–Whitney *U*-test. MenC: low *N* = 25, high *N* = 27; MenW: low *N* = 46, high *N* = 35; and MenY: low *N* = 40, high *N* = 34. ****p < 0.0001.

The functional antibody titers of the low and high vaccine responders 28 days postvaccination are depicted in Figure 2A. Although the low responders possess a functional antibody titer below or matching the first quartile of that of the total group, most of these values were above the protection level (functional antibody titer of 8). The fold differences in functional antibody titers between the low and high vaccine responders at 28 days postvaccination is 141.5, 15.2, and 16.7, for MenC, MenW, and MenY, respectively. No difference in prevaccination functional antibody titers was found between the low and high responders (Figure 2B). The functional antibody titers of part of the low vaccine responders had declined below the protection level at 1-year postvaccination, whereas all high responders were still highly protected (Figure 2C).

Participant characteristics were compared between the low and high vaccine responders (Table 1). Only for MenC, the low responders were significantly older as compared with the high responders. All participants possessed prevaccination TT-specific antibodies. Sex distribution, the number of CMV seropositive participants, and BMI were comparable between the low and high responders. Moreover, no significant differences in disease incidence (within the last year), medication use (within the last 6 months), the incidence of recent infections (within the last 4 weeks), smoking, or physical activity were observed. Not all participants were identified as low or high responder consistently for all meningococcal groups.

**Table 1:** Participant characteristics.

	MenC		MenW		MenY	
	Low	High	Low	High	Low	High

	*N* = 25	*N* = 27	*N* = 46	*N* = 35	*N* = 40	*N* = 34
Age (95% CI)	59.1* (57.5–60.7)	56.1* (54.5–57.7)	58.3 (57.1–59.6)	56.9 (55.5–58.4)	57.7 (56.4–59.1)	56.6 (55.0–58.1)
Males (%)	13 (52%)	16 (59%)	21 (45.6%)	19 (54.3%)	20 (50%)	17 (50%)
CMV seropositive (%)	10 (40%)	17 (63%)	21 (45.6%)	18 (51.4%)	15 (37.5%)	17 (50%)
BMI (95% CI)	24.9 (23.5–26.3)	24.8 (23.5–26.2)	25.2 (24.2–26.4)	25.8 (24.7–27.0)	24.8 (23.7–26.0)	26.5 (25.2–27.8)
TT-specific prevaccination IgG Geomean (95% CI)	0.72 (0.47–1.11)	1.20 (0.77–1.88)	0.84 (0.5–1.28)	0.87 (0.55–1.37)	1.08 (0.76–1.53)	0.78 (0.51–1.17)
**Disease in last year (number, %)**						
Diabetes type II	0 (0%)	1 (3.7%)	2 (4.3%)	3 (8.6%)	1 (2.5%)	2 (5.9%)
High blood pressure	5 (20.0%)	3 (11.1%)	8 (17.4%)	5 (14.3%)	6 (15%)	8 (23.5%)
Vascular diseases	0 (0%)	0 (0%)	0 (0%)	0 (0%)	1 (2.5%)	0 (0%)
Lung diseases	1 (4.0%)	1 (3.7%)	0 (0%)	4 (11.4%)	2 (5%)	2 (5.9%)
Rheumatic diseases	1 (4.0%)	1 (3.7%)	2 (4.3%)	0 (0%)	2 (5%)	0 (0%)
Gastro-intestinal diseases	0 (0%)	2 (7.4%)	2 (4.3%)	1 (2.9%)	2 (5%)	2 (5.9%)
Other diseases	1 (4.0%)	1 (3.7%)	1 (2.2%)	3 (8.6%)	1 (2.5%)	3 (8.8%)
No serious disease	18 (72%)	19 (70.4%)	35 (76.1%)	26 (74.3%)	31 (77.5%)	24 (70.6%)
**Medication last 6 months (number, %)**						
Medication for infection^a^	0 (0%)	4 (14.8%)	3 (6.5%)	4 (11.4%)	2 (5%)	2 (5.9%)
Cholesterol lowering medication	2 (8.0%)	2 (7.4%)	5 (10.9%)	5 (14.3%)	3 (7.5%)	4 (11.8%)
Diabetic medication	0 (0%)	1 (3.7%)	1 (2.2%)	3 (8.6%)	0 (0%)	2 (5.9%)
Blood pressure lowering medication	7 (28.0%)	3 (11.1%)	9 (19.6%)	6 (17.1%)	7 (17.5%)	8 (23.5%)
Immunosuppressive medication	0 (0%)	1 (3.7%)	1 (2.2%)	2 (5.7%)	1 (2.5%)	1 (2.9%)
No medication	18 (72%)	19 (70.4%)	32 (69.6%)	23 (65.7%)	30 (75%)	23 (67.6%)
**Infections (number, %)**						
Influenza < 4 weeks	0 (0%)	1 (3.7%)	1 (2.2%)	0 (0%)	1 (2.5%)	0 (0%)
Cold < 4 weeks	6 (24.0%)	3 (11.1%)	7 (15.2%)	10 (28.6%)	7 (17.5%)	6 (17.6%)
No infection < 4 weeks	19 (76.0%)	25 (92.6%)	39 (85.8%)	26 (74.3%)	33 (82.5%)	29 (85.3%)
**Smoking (number, %)**						
Cigarette smoking	6 (24.0%)	3 (11.1%)	6 (13%)	3 (8.6%)	6 (15%)	4 (11.8%)
Cigars, pipe smoking	1 (4.0%)	0 (0%)	3 (6.5%)	1 (2.9%)	3 (7.5%)	1 (2.9%)
No smoking	18 (72%)	25 (92.6%)	38 (82.6%)	32 (91.4%)	32 (80%)	30 (88.2%)
**Physical activity (number, %)**						
Weekly or more	17 (68.0%)	19 (70.4%)	31 (67.4%)	24 (68.6%)	27 (67.5%)	25 (73.5%)
Less than weekly	4 (16.0%)	2 (7.4%)	8 (17.4%)	4 (11.4%)	6 (15%)	4 (11.8%)
No activity	4 (16.0%)	7 (25.9%)	7 (15.2%)	8 (22.9%)	7 (17.5%)	6 (17.6%)

*^a^Medication used more than 3 months ago and mainly consisting of corticosteroids and antibiotics*.

*p < 0.05, significant differences are depicted in bold and underlined. The chi-square test was used to determine statistical significances.

### Differences in Prevaccination Immune Markers between High and Low Vaccine Responders

**Figure 3:**
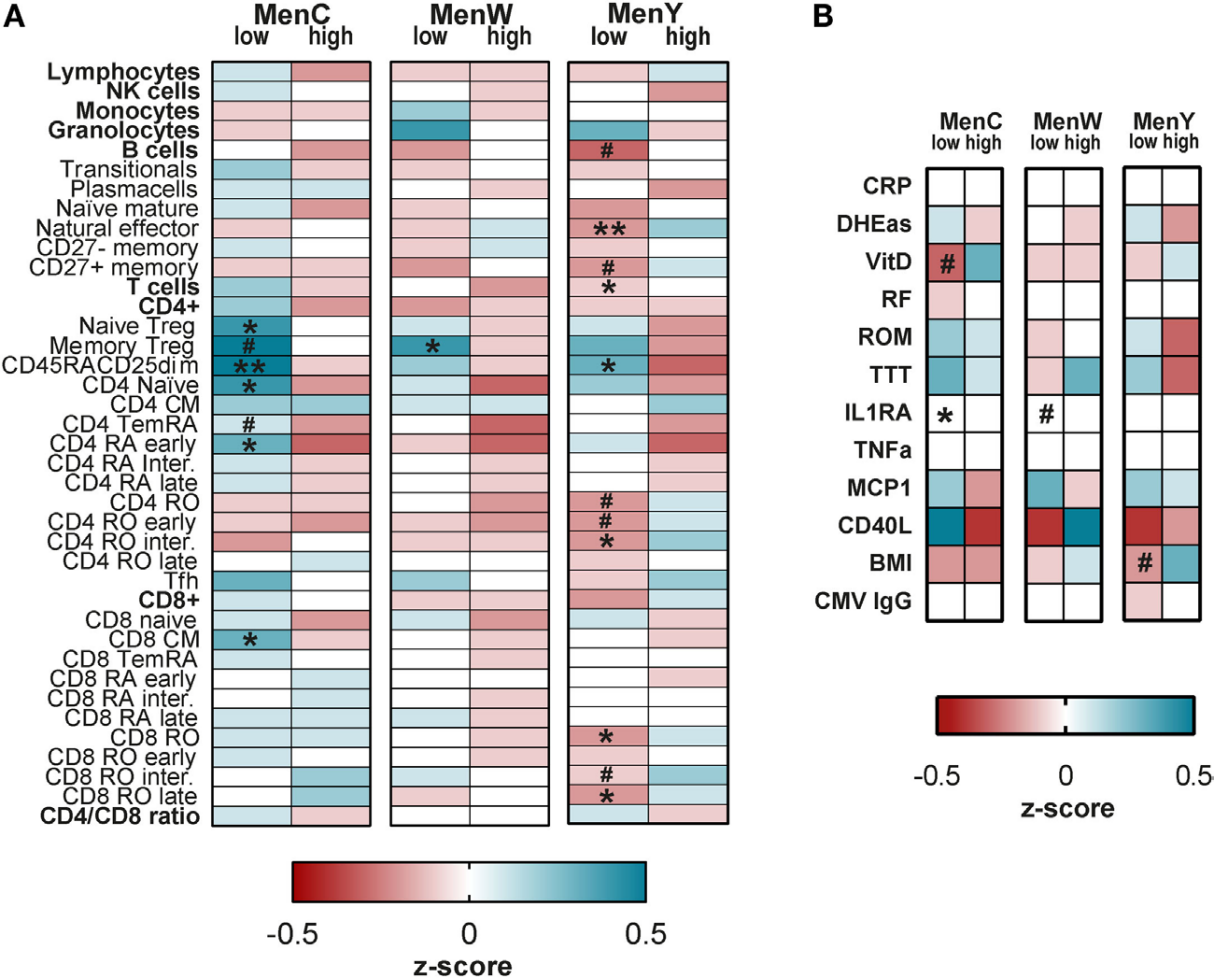
Comparison of prevaccination immune markers between the high and low vaccine responders. Heat maps comparing the absolute immune cells numbers **(A)**, serum cytokines and biochemical markers **(B)** between the low and high vaccine responders for the different meningococcal serotypes separately. The absolute immune cell numbers as well as the concentrations of the serum markers were normalized to *z*-scores using the geometric means. The geometric means of the two groups were compared with the overall group geometric mean and standard deviation. The normalized *z*-scores are displayed on a color scale, ranging from red (below the geometric mean of the total group) to blue (above the geometric mean of the total group). The white color indicates values that are equal to the group geometric mean. The stronger the deviation from the group geometric mean, the darker the color. The different immune markers were compared between the low and high responders using the Mann–Whitney *U*-test. ^#^*p* < 0.1, *p < 0.05, **p < 0.01. MenC: low *N* = 25, high *N* = 27; MenW: low *N* = 46, high *N* = 35; and MenY: low *N* = 40, high *N* = 34.

At first, the absolute numbers of immune cells were compared between the high and low responders for the different meningococcal groups separately (Figure 3A). Low responders to MenC possessed significantly higher absolute numbers of naïve Treg (*p* = 0.033), CD45RA^+^CD25^dim^ (*p* = 0.005), CD4 naïve (*p* = 0.021), CD4 TemRA early (*p* = 0.024), and CD8 CM (*p* = 0.038) cells as compared with the high responders (Figure 3A; Figures S1A,C,D,F,G in Supplementary Material). Moreover, trends toward higher absolute numbers of memory Treg (*p* = 0.084) and CD4 TemRA (*p* = 0.057) cells were found in the low responders (Figure 3A; Figures S1B,E in Supplementary Material). In addition, the low responders to MenC showed lower serum levels of IL-1Ra (*p* = 0.035) as compared with the high responders (Figure 3B; Figures S1H,I in Supplementary Material), as well as a trend toward lower VitD levels (*p* = 0.075) (Figure 3B; Figure S1J in Supplementary Material).

Low responders for MenW possessed significantly higher absolute numbers of memory Treg cells (*p* = 0.039) (Figure 3A; Figure S2A in Supplementary Material) as well as a trend toward lower levels of IL-1Ra (*p* = 0.057) (Figure 3B; Figure S2B in Supplementary Material) as compared with the high responders.

Finally, the low responders for MenY had significantly higher absolute numbers of CD45RA^+^CD25^dim^ cells (*p* = 0.022) as well as lower absolute numbers of natural effector (CD27^+^IgD^+^) B cells (*p* = 0.008), T cells (*p* = 0.043), CD4 TemRO intermediate cells (*p* = 0.011), CD8 TemRO cells (*p* = 0.027), and CD8 TemRO late cells (*p* = 0.036) than the high responders (Figure 3A; Figures S3B,D,E,H,I,K in Supplementary Material). Moreover, trends toward lower absolute numbers of total B cells (*p* = 0.060), CD27^+^memory B cells (Bmem) (*p* = 0.062), CD4RO T cells (*p* = 0.070), CD4RO early T cells (*p* = 0.081), and CD8 TemRO intermediate T cells (*p* = 0.090) (Figure 3A; Figures S3A,C,F,G,J in Supplementary Material) as well as a trend to a lower BMI (*p* = 0.053) (Figure 3B; Figure S3I in Supplementary Material) were observed in the low responders.

### Multivariate RDA Revealing Significant Associations between the Prevaccination Immune Phenotype and Vaccine Responsiveness

**Figure 4:**
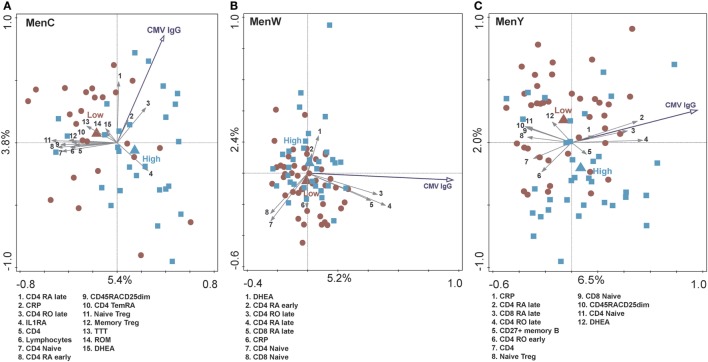
Redundancy analysis (RDA) assessing the association between the prevaccination immune phenotype and the meningococcal vaccine response. RDA of samples collected 28 days postvaccination for MenC **(A)**, MenW **(B)**, and MenY **(C)**. Low vaccine responders are shown in red circles; High responders are shown in light blue squares. The first and second ordination axes are plotted, including the percentages of explained variation. Overall, 14.5, 10.4, and 12.0% of the variation in the datasets was explained for MenC, MenW, and MenY, respectively. The vaccine response variable (i.e., low or high responder) was significantly associated with the immune phenotype for MenC (*p* = 0.012, FDR = 0.07) and MenY (*p* = 0.028, FDR = 0.098), but not for MenW (*p* = 0.068, FDR = 0.16). CMV was significantly associated with the immune composition for MenW (*p* = 0.002, FDR = 0.014) and MenY (*p* = 0.002, FDR = 0.014) but not for MenC (*p* = 0.098, FDR = 0.23). Other environmental variables tested (i.e., BMI, age, and sex) did not significantly influence the variation in the dataset. The biological variables with the highest variation explained by the explanatory variables are depicted in the plots (FitE > 15). The length of the arrows relates to the strength of the association.

In order to determine whether high or low vaccine responsiveness at day 28 postvaccination was significantly associated with all measured prevaccination immune markers combined, hereafter called the immune phenotype; a multivariate RDA was performed for the three meningococcal groups separately (Figures 4A–C). Overall the included variables (age, sex, CMV, BMI, and vaccine response) explained 14.5, 10.4, and 12.0% of the total variation in immune phenotype for MenC, MenW, and MenY, respectively.

For MenC and MenY, the variable “vaccine response” was significantly associated with the immune phenotype (MenC: *p* = 0.012, FDR = 0.07 and MenY: *p* = 0.028, FDR = 0.098) (Figures 4A,C). As expected, based on the heat map depicted in Figure 3A, for MenW no significant association between vaccine response and immune phenotype was observed (*p* = 0.068, FDR = 0.16) (Figure 4B).

For MenC group, higher levels of DHEA, TTT, and ROM as well as higher absolute numbers of memory Treg cells, naïve Treg cells, CD45RA^+^CD25^dim^ cells, CD4 TemRA early cells, CD4 naïve cells, lymphocytes, and CD4 T cells were strongly associated with low responsiveness, whereas higher levels of IL-1Ra were related with high responsiveness (Figure 4A). For MenY group, higher levels of DHEA and higher absolute numbers of CD4 naïve, CD45RA^+^CD25^dim^, CD8 naïve, and naïve Treg cells were strongly associated with low vaccine responsiveness, while high absolute numbers of Bmem cells were linked to high vaccine responsiveness (Figure 4C).

In addition, CMV seropositivity was significantly associated with the immune phenotype (Figure 4A: *p* = 0.098, FDR = 0.23, Figure 4B, *p* = 0.002, FDR = 0.014, and Figure 4C, *p* = 0.002, FDR = 0.014). In these analyses, CMV seropositivity was associated with higher absolute numbers of CD4RO late, CD4RA late, and CD8RA late T cells, and not related to either low or high vaccine responsiveness. Of note, the explanatory variables BMI, age, and sex were not significantly associated with the immune phenotype.

### Differences in Immune Cell Ratios in the CD4 T Cell Compartment between the High and Low Vaccine Responders

**Figure 5:**
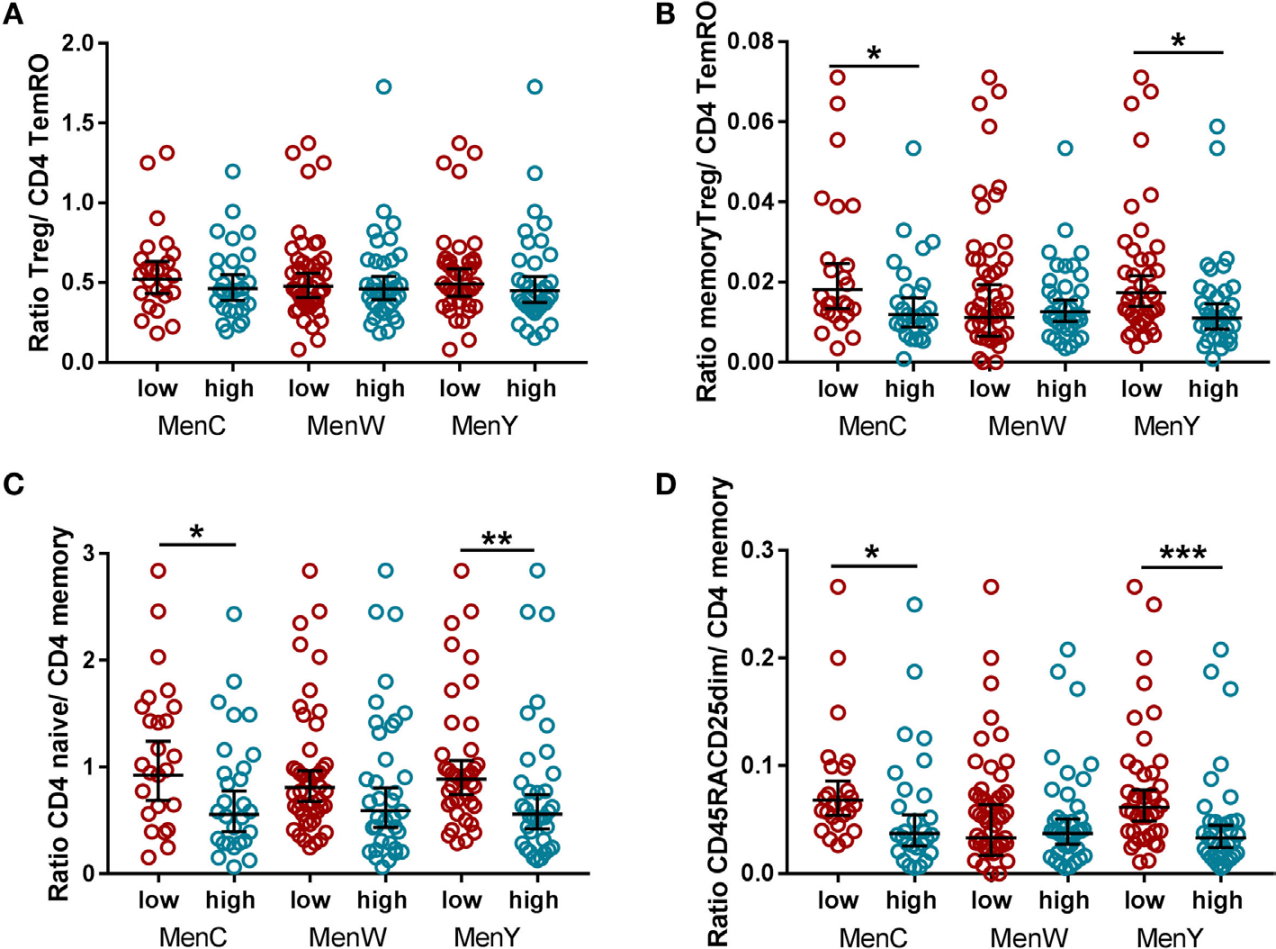
Comparison of immune cell ratios in the CD4 T cell compartment between the high and low vaccine responders. Comparison of the Treg/TemRO ratio **(A)**, memory Treg/TemRO ratio **(B)**, CD4 naïve/CD4 memory ratio **(C)**, and CD45RA^+^CD25^dim^/CD4 memory ratio **(D)** between the low (red) and high (blue) vaccine responders for the different meningococcal groups separately. The total CD4 memory cells were determined as the sum of the CD4 CM, CD4 TemRO, and CD4 TemRA cells. The low and high responders were compared with the Mann–Whitney *U*-test. *p < 0.05, **p < 0.01, and ***p < 0.001.

In relation to the mentioned findings of higher naïve CD4 T cells and memory Treg cells in the low vaccine responders, we determined whether the ratio of naïve to memory cells, as well as the ratio of Treg to effector cells in the CD4 T cell compartment was different between the low and high vaccine responders (Figure 5). No difference in the ratio between the total Treg cells and the number of CD4 TemRO T cells was observed (Figure 5A), whereas the ratio of memory Treg cells to CD4 TemRO T cells was significantly higher in the low responders for MenC and MenY as compared with the high responders (Figure 5B). In addition, the low responders for MenC and MenY possessed a significantly higher ratio of the naïve CD4 T cells and the total CD4 memory cells (Figure 5C), as well as a higher ratio of the post thymically expanded CD45RA^+^CD25^dim^ cells and the total CD4 memory cells (Figure 5D). Thus, the higher numbers of both naïve CD4 T cell subsets and Treg cells as seen in low responders at baseline (prevaccination) give rise to clear compositional changes in the peripheral CD4 T cell compartment.

## Discussion

In this explorative study, we investigated whether the prevaccination immune phenotype was significantly different between the middle-aged adults being either low or high responder after a primary meningococcal vaccination. Interestingly, the numbers of several CD4 T cell subsets differed between the low and high vaccine responders. More specifically, low vaccine responders possessed higher numbers of naïve Treg, memory Treg, naïve CD4 cells, and the subset of post thymically expanded CD4^+^CD45RA^+^CD25^dim^ T cells, whereas high responders showed high levels of serum IL-1Ra. These results suggest that the prevaccination CD4 signature may be used to identify middle-aged adults who are potential non/low responders to a primary meningococcal vaccine. Identification of these middle-aged adults may help improve timely vaccination strategies, since vaccination schemes, doses, and adjuvant use might be adapted to improve the vaccine responsiveness in these adults.

Numbers of Treg cells are known to increase with advancing age and suggest elevated immune suppression in older adults, although the exact functionality of these Treg cells in aging individuals is still under investigation (19, 48). Accordingly, high numbers of Treg cells were previously associated with low VZV vaccine responses in nursing home elderly (18). Within our study, high absolute numbers of both naïve and memory Treg cells were associated with low vaccine responsiveness. Naïve Treg cells express CCR7 enabling these cells to migrate to lymphoid organs, whereas memory Treg cells home to the sites of inflammation along with effector T cells (49, 50). Accordingly, our results suggest enhanced suppression of the vaccine response both in the lymphoid organs as well as the site of vaccination in low responders. Elevated numbers of Treg cells might suppress T cell responses toward the tetanus carrier in this conjugated meningococcal vaccine and/or inhibit B cell responses directly (51). Our results confirm previous findings of high numbers of memory Treg cells in low vaccine responders to influenza (21), whereas we are the first showing an association between low vaccine responsiveness and high numbers of naïve Treg cells. Of importance, we observed a higher ratio of memory Treg to effector CD4 T cells in the low responders, suggesting a shift in the Treg/Teffector balance, as previously observed with advancing age (21).

The consistent association found between high numbers ofCD4 naïve and the post thymically expanded CD4^+^CD45RA^+^CD25^dim^ cells and a higher ratio of these naïve cells to the memory CD4 T cell compartment with low vaccine responsiveness was unexpected, since a naïve T cell repertoire is generally accepted to be beneficial in older adults (9, 11). Previously, the CD4^+^CD45RA^+^CD25^dim^ subset was found to represent a broad and functional reservoir of naïve CD4 T cells, although some contraction in certain TCR Vβ families was observed in comparison to naïve CD4 T cells that recently left the thymus (52). Since no prior studies were available that linked the numbers of CD4^+^CD45RA^+^CD25^dim^ cells to vaccine responses, our findings indicate the necessity for further research into repertoire size and functionality of these cells. However, the increase in memory Treg cells might be the dominant factor in predicting vaccine response, overruling the presence of a capable naïve CD4 T cell repertoire.

In line with the different studies investigating biomarkers for influenza and hepatitis B vaccine responses in the elderly (17, 23, 25), we observed trends toward lower numbers of switched memory CD27^+^ B cells in the low responders. In contrast, we did not find increased numbers of CD27-memory B cells in the low responders, as reported by others in vaccine recipients aged over 65 years (23). In addition, low numbers of natural effector CD27^+^IgD^+^ B cells that were previously found to decrease with age (24) were observed in the low vaccine responders. Since we previously described that IgM is essential in the antibody functionality against the meningococcal groups (4), lower numbers of these natural effector cells, mainly producing IgM, likely explain the lower functional antibody titers.

Currently, effects of latent CMV infection on vaccine responses are controversial (53). Despite the clear associations between CMV seropositivity and higher numbers of late differentiated T cells, we did not find an association between CMV seropositivity and meningococcal vaccine responsiveness. As this meningococcal vaccine response is primarily B cell mediated, the effect of CMV might be limited. Hence, the effects of CMV on T cell-mediated vaccine responses, i.e., to influenza and VZV vaccination should be further elucidated. Although frequently suggested by others (54–57), we did not observe any effects of sex and BMI on the vaccine responses. Moreover, the effect of chronological age was inconsistent, although low responders to MenC were significantly older than high responders.

Of note, high levels of IL-1Ra were found in the high responders. IL-1Ra is known as the receptor antagonist of the IL-1 family, executing anti-inflammatory functions (58, 59). These results possibly suggest that IL-1Ra acts as an anti-inflammatory counterpart of the “inflammageing” process. Of note, a trend toward high levels of MCP-1 was found in the low responders. MCP-1 is classified as a pro-inflammatory cytokine, attracting monocytes to the site of inflammation, and serum levels were shown to increase with age (60). Consequently, our findings may suggest a higher pro-inflammatory state in the low responders (33, 34). Nevertheless, serum levels of other inflammatory cytokines, such as IL-6 and TFN-α were still low in all participants. Remarkably, trends toward higher levels of sCD40L were found in the low responders for MenC, as compared with lower levels in the low responders for MenW and MenY, which needs further evaluation.

Of importance, associations with the immune phenotype were primarily found between the extremes in the vaccine response, being either low or high responders. The intermediate group showed high variability of immune markers. In addition, our results may imply meningococcal group-specific associations between the prevaccination immune phenotype and vaccine responsiveness. Noteworthy, the difference in functional antibody titers between the low and high vaccine responders was largest for MenC, possibly explaining the higher numbers of immune parameters found associated with the vaccine response for this meningococcal group. Moreover, the participants that were classified as low or high responder did not completely overlap between the different meningococcal groups. This might be explained by the structural differences in the meningococcal polysaccharides by which different meningococcal epitopes will induce distinct immune responses (61), also shown previously for several pneumococcal conjugated polysaccharides (62). Possible structural differences will affect the B cell processing and subsequently the quality and quantity of the T cell help provided by the carrier, that might cause differences in antibody functionality to the various polysaccharides. Currently, differences between meningococcal group-specific polysaccharides conjugated to TT are not known (63). Furthermore despite similar prevaccination functional antibody titers in the low and high vaccine responders, differences in numbers of meningococcal group-specific memory B cells in the bone marrow could have been present due to historical contacts (64) and affect the vaccine response. Nevertheless, the finding of meningococcal group-specific associations between vaccine responsiveness and immune phenotype is remarkable and requires further research. Of note, we previously found that most participants possessed high prevaccination TT-specific antibody levels (4). Importantly, no direct correlation between the antibody responses to TT or the different polysaccharides and the strength or classification of the T cell response induced by the TT-carrier protein was observed (5). In this study, the similar prevaccination TT-specific antibody levels in the low and high responders, suggests that the prevaccination immunity against the TT carrier protein did not largely affect the immune responses.

An important strength of this study is the ability to compare multiple antigens and multiple immune parameters within the same group of participants. Also, the primary nature of the vaccination allowed us to explore the use of biomarkers, without the strong interference of prevaccination meningococcal immunity, as often seen in other studies. Unfortunately, the presence of prevaccination immunity in some participants did interfere with the long-term functional antibody titers (after 1 year). Consequently, we were not able to investigate the associations between prevaccination immune phenotype and the long-term vaccine responsiveness, since exclusion of participants with detectable prevaccination functional antibody titers dramatically reduced the power of the statistical analysis. In addition, information on the genetic background of the participants could have added to the predictive factors in our analysis, since several studies found associations between genetic signatures and vaccine responsiveness (25, 31, 32).

Future studies, analyzing large cohorts, using different vaccines, and using similar biomarker analyzing techniques are warranted to validate the use of the suggested biomarkers. Hence, systems vaccinology, combining data on genetic background, and environmental factors such as diet, stress, and infections, and even microbiome composition is a promising tool to discover these predictive biomarkers (65). Moreover, future research should compare the suitability of biomarkers in cohorts of different ages, in order to determine the predictive values of these markers over the entire lifespan.

In conclusion, our explorative biomarker analysis suggests several associations between the prevaccination immune phenotype and vaccine responsiveness after primary meningococcal vaccination in middle-aged adults. In general, an altered CD4 T cell signature, involving high absolute numbers of naïve Treg, memory Treg, naïve CD4 T cells, and CD45RA^+^CD25^dim^ T cells might be used as a predictive immune phenotype for low vaccine responsiveness in middle-aged adults. Accordingly, these findings support the development of vaccination strategies to enhance the memory immunity before reaching old age, in the rapidly aging population.

## Ethics Statement

Written informed consent was obtained from all participants prior to enrollment and all procedures were in accordance with the Declaration of Helsinki. The medical ethical committee: Medical Research Ethics Committees United (MEC-U) approved the study and the study was registered at the Dutch trial register (Protocol no. NTR4636).

## Author Contributions

MH, GB, MZ, A-MB, and AB conceptualized the study. MH planned and performed the clinical work and executed the laboratory experiments. MH and SF performed the statistical analysis. MH, GB, MZ, MD, SF, A-MB, and AB interpreted the data and wrote the manuscript. All authors critically revised the manuscript.

## Conflict of Interest Statement

MH, GB, SF, MZ, and A-MB declare no conflict of interest. AB is a consultant for Grunenthal Gmbh (Germany).
