# Supplementary material for: An Explorative Biomarker Study for Vaccine Responsiveness after a Primary Meningococcal Vaccination in Middle-Aged Adults
Source: Front Immunol. 2018 Jan 11;8:1962. doi: 10.3389/fimmu.2017.01962 (PMC5768620; doi:10.3389/fimmu.2017.01962)
Supplement: Supplementary file 1 [file Table_1.DOCX]

**Supplementary Table 1**. Phenotypical definitions of the immune cells subsets

| **Subset** | **Phenotype definition** |
| --- | --- |
| Monocytes | SSC^inter^CD45+ |
| Granulocytes | SSC^high^CD45+ |
| Lymphocytes | SSC^low^CD45+ |
| NK cells | SSC^low^CD45+CD3-CD16+ or CD56+ |
| B-cells | SSC^low^CD45+CD19+ |
| Translational | SSC^low^CD45+CD27-CD38+ |
| Plasma cells | SSC^low^CD45+CD27+CD38+ |
| Naïve mature | SSC^low^CD45+CD27-IgD+CD38^dim^ |
| Natural effector | SSC^low^CD45+CD27+IgD+CD38^dim^ |
| CD27- memory | SSC^low^CD45+CD27-IgD-CD38^dim^ |
| CD27+ memory | SSC^low^CD45+CD27+IgD-CD38^dim^ |
| T-cells | SSC^low^CD45+CD3+ |
| CD4 T-cells | SSC^low^CD45+CD3+CD4+ |
| Naïve Tregs | SSC^low^CD45+CD3+CD4+CD45RO-CD25^int^ |
| Memory Tregs | SSC^low^CD45+CD3+CD4+CD45RO+CD25^high^ |
| CD45RA+CD25dim | SSC^low^CD45+CD3+CD4+CD45RO-CD25^dim^ |
| CD4 naïve | SSC^low^CD45+CD3+CD4+CD45RO-CCR7+ |
| CD4 CM | SSC^low^CD45+CD3+CD4+CD45RO+CCR7+ |
| CD4 TemRA | SSC^low^CD45+CD3+CD4+CD45RO-CCR7- |
| CD4 TemRA early | SSC^low^CD45+CD3+CD4+CD45RO-CCR7-CD27+CD28+ |
| CD4 TemRA intermediate | SSC^low^CD45+CD3+CD4+CD45RO-CCR7-CD27-CD28+ |
| CD4 TemRA late | SSC^low^CD45+CD3+CD4+CD45RO-CCR7-CD27-CD28- |
| CD4 TemRO | SSC^low^CD45+CD3+CD4+CD45RO+CCR7- |
| CD4 TemRO early | SSC^low^CD45+CD3+CD4+CD45RO+CCR7-CD27+CD28+ |
| CD4 TemRO intermediate | SSC^low^CD45+CD3+CD4+CD45RO+CCR7-CD27-CD28+ |
| CD4 TemRO late | SSC^low^CD45+CD3+CD4+CD45RO+CCR7-CD27-CD28- |
| Tfh | SSC^low^CD45+CD3+CD4+CXCR5+ |
| CD8 T-cells | SSC^low^CD45+CD3+CD8+ |
| CD8 naïve | SSC^low^CD45+CD3+CD8+CD45RO-CCR7+ |
| CD8 CM | SSC^low^CD45+CD3+CD8+CD45RO+CCR7+ |
| CD8 TemRA | SSC^low^CD45+CD3+CD8+CD45RO-CCR7- |
| CD8 TemRA early | SSC^low^CD45+CD3+CD8+CD45RO-CCR7-CD27+CD28+ |
| CD8 TemRA intermediate | SSC^low^CD45+CD3+CD8+CD45RO-CCR7-CD27+CD28- |
| CD8 TemRA late | SSC^low^CD45+CD3+CD8+CD45RO-CCR7-CD27-CD28- |
| CD8 TemRO | SSC^low^CD45+CD3+CD8+CD45RO+CCR7- |
| CD8 TemRO early | SSC^low^CD45+CD3+CD8+CD45RO+CCR7-CD27+CD28+ |
| CD8 TemRO intermediate | SSC^low^CD45+CD3+CD8+CD45RO+CCR7-CD27+CD28- |
| CD8 TemRO late | SSC^low^CD45+CD3+CD8+CD45RO+CCR7-CD27-CD28- |
