# Supplementary material for: An Explorative Biomarker Study for Vaccine Responsiveness after a Primary Meningococcal Vaccination in Middle-Aged Adults
Source: Front Immunol. 2018 Jan 11;8:1962. doi: 10.3389/fimmu.2017.01962 (PMC5768620; doi:10.3389/fimmu.2017.01962)
Supplement: Supplementary file 2 [file Image_1.PDF]

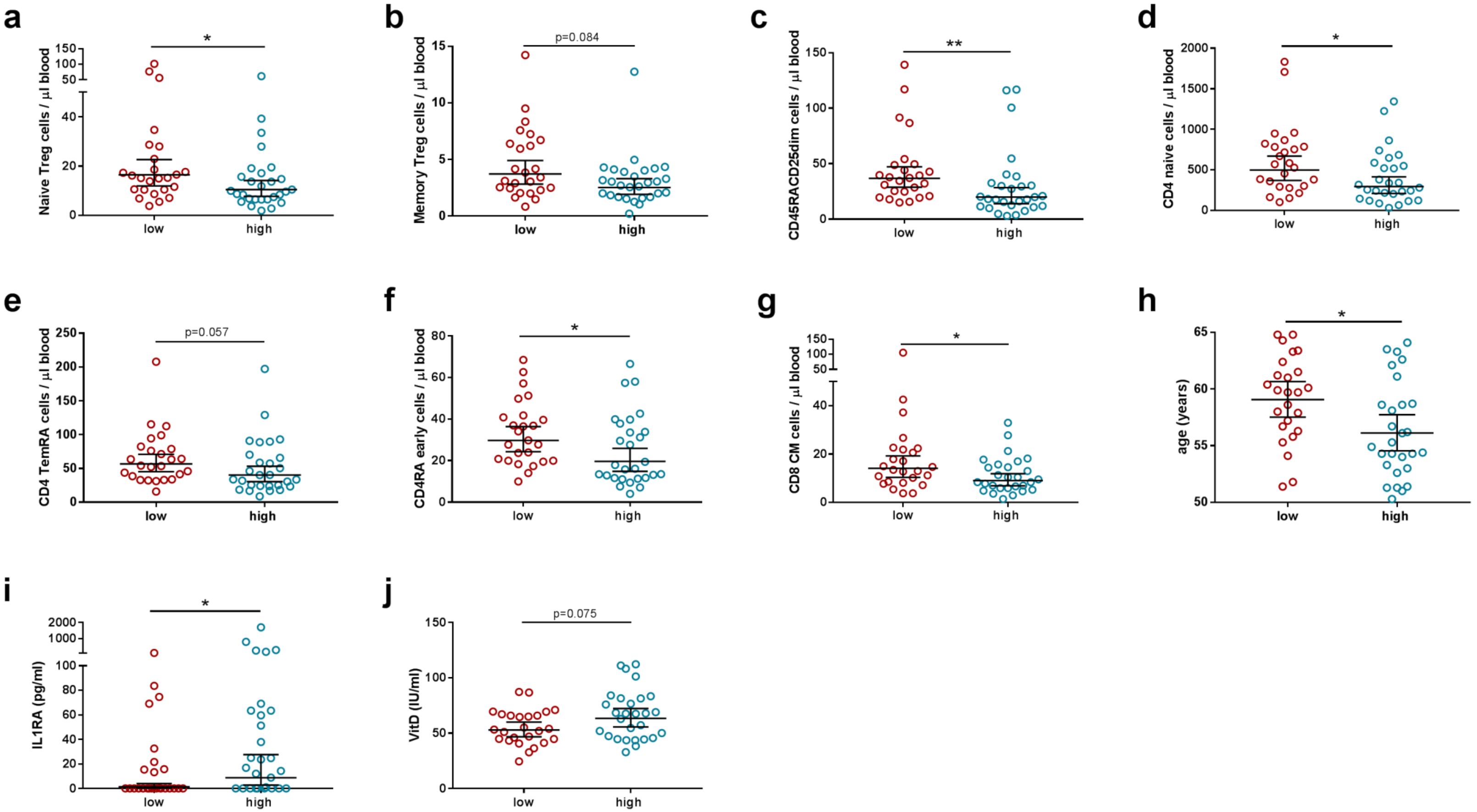

Supplementary Figure 1. Analysis of differences in immune markers between high and low responders for MenC.
The differences in absolute numbers of naïve Treg **(a)**, Memory Treg **(b)**, CD45RA+CD25dim **(c)**, CD4 naïve **(d)**, CD4 TemRA **(e)**, CD4 TemRA early **(f)**, CD8 CM **(g)**, age **(h)**, and the levels of IL1Ra **(i)**, and VitD **(j)** between the low (red, N=25) and high (blue, N=27) responders for MenC. The Geometric means with 95% CI interval are indicated in the graphs. The low and high responders were compared for the different immune markers using the Mann Whitney U test. Trends are given as p-values. * p<0.05, ** p<0.01.
