# Supplementary material for: An Explorative Biomarker Study for Vaccine Responsiveness after a Primary Meningococcal Vaccination in Middle-Aged Adults
Source: Front Immunol. 2018 Jan 11;8:1962. doi: 10.3389/fimmu.2017.01962 (PMC5768620; doi:10.3389/fimmu.2017.01962)
Supplement: Supplementary file 3 [file Image_2.PDF]

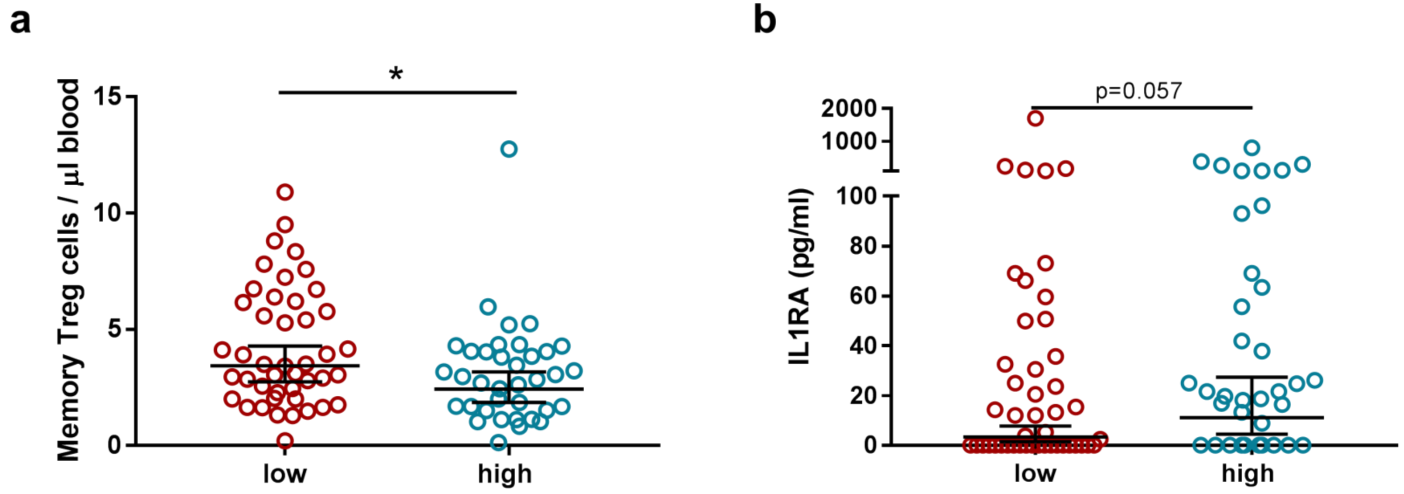

Supplementary Figure 2. Analysis of differences in immune markers between the high and low responders for MenW .
The difference in absolute numbers of memory Treg **(a)** and levels of IL1Ra **(b)** between the low (red, N= 46) and high (blue, N=35) responders for MenW. The geometric mean with 95% CI interval is indicated in the graphs. The early low and high responders were compared for the different immune markers using the Mann Whitney U test. Trends are given as p-values. * $p<0.05$.
