# Supplementary material for: An Explorative Biomarker Study for Vaccine Responsiveness after a Primary Meningococcal Vaccination in Middle-Aged Adults
Source: Front Immunol. 2018 Jan 11;8:1962. doi: 10.3389/fimmu.2017.01962 (PMC5768620; doi:10.3389/fimmu.2017.01962)
Supplement: Supplementary file 4 [file Image_3.PDF]

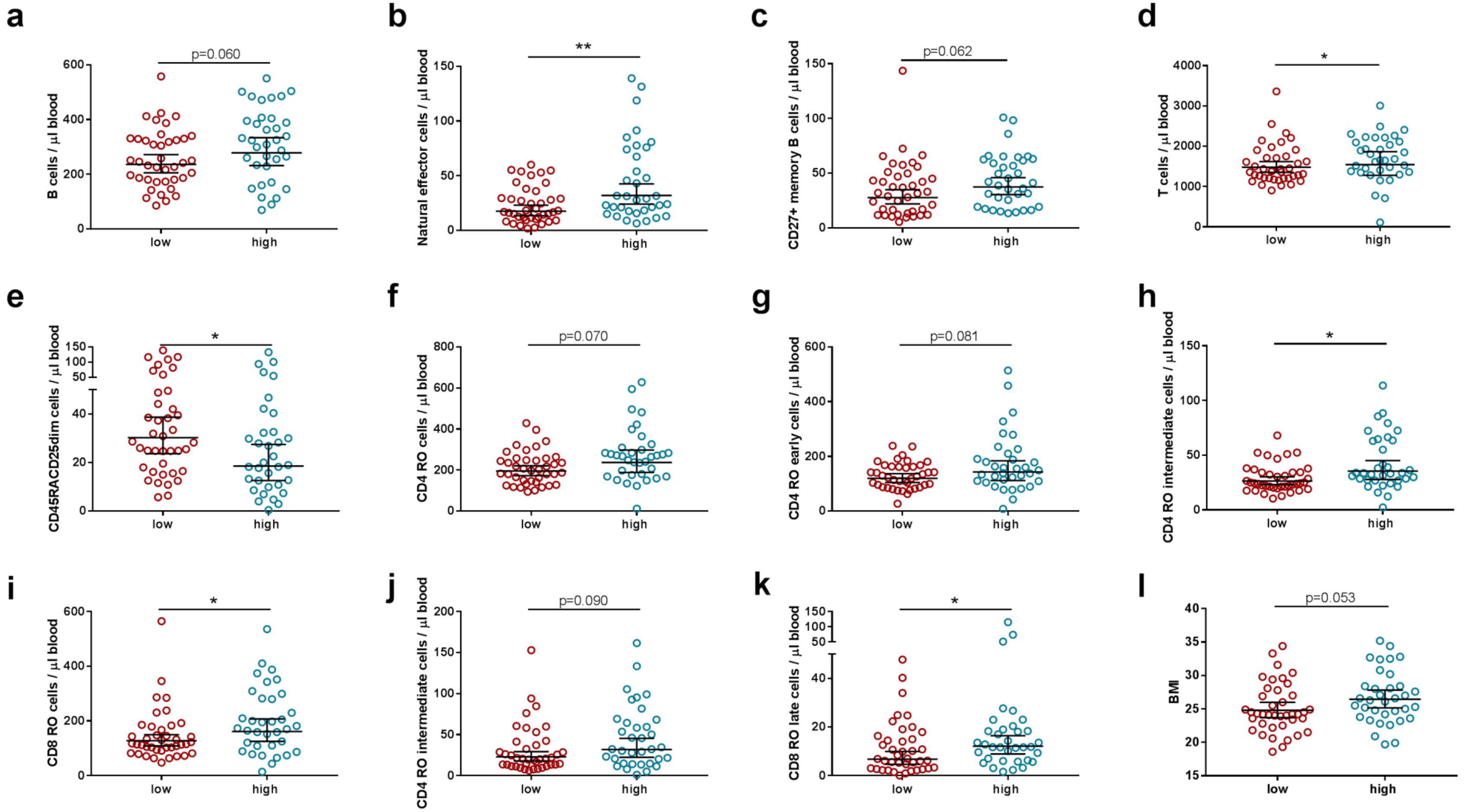

Supplementary Figure 3. Analysis of differences in immune markers between the high and low responders for MenY.
The difference in absolute numbers of B cells **(a)**, Natural effector B-cells **(b)**, CD27+ memory B-cells **(c)**, T-cells **(d)**, CD45RA+CD25dim cells **(e)**, CD4 TemRO cells **(f)**, CD4 TemRO early cells **(g)**, CD4 TemRO intermediate cells **(h)**, CD8 TemRO cells **(i)**, CD4 TemRO intermediate cells **(j)**, CD8 TemRO late cells **(k)**, and BMI **(l)** between the low (red, N= 40) and high (blue, N= 34) responders for MenY. The geometric mean with 95% CI interval is indicated in the graphs. The low and high responders were compared for the different immune markers using the Mann Whitney U test. Trends are given as p-values. * p<0.05. ** p<0.01.
