# Supplementary material for: An Explorative Biomarker Study for Vaccine Responsiveness after a Primary Meningococcal Vaccination in Middle-Aged Adults
Source: Front Immunol. 2018 Jan 11;8:1962. doi: 10.3389/fimmu.2017.01962 (PMC5768620; doi:10.3389/fimmu.2017.01962)
Supplement: Supplementary file 5 [file Image_4.PDF]

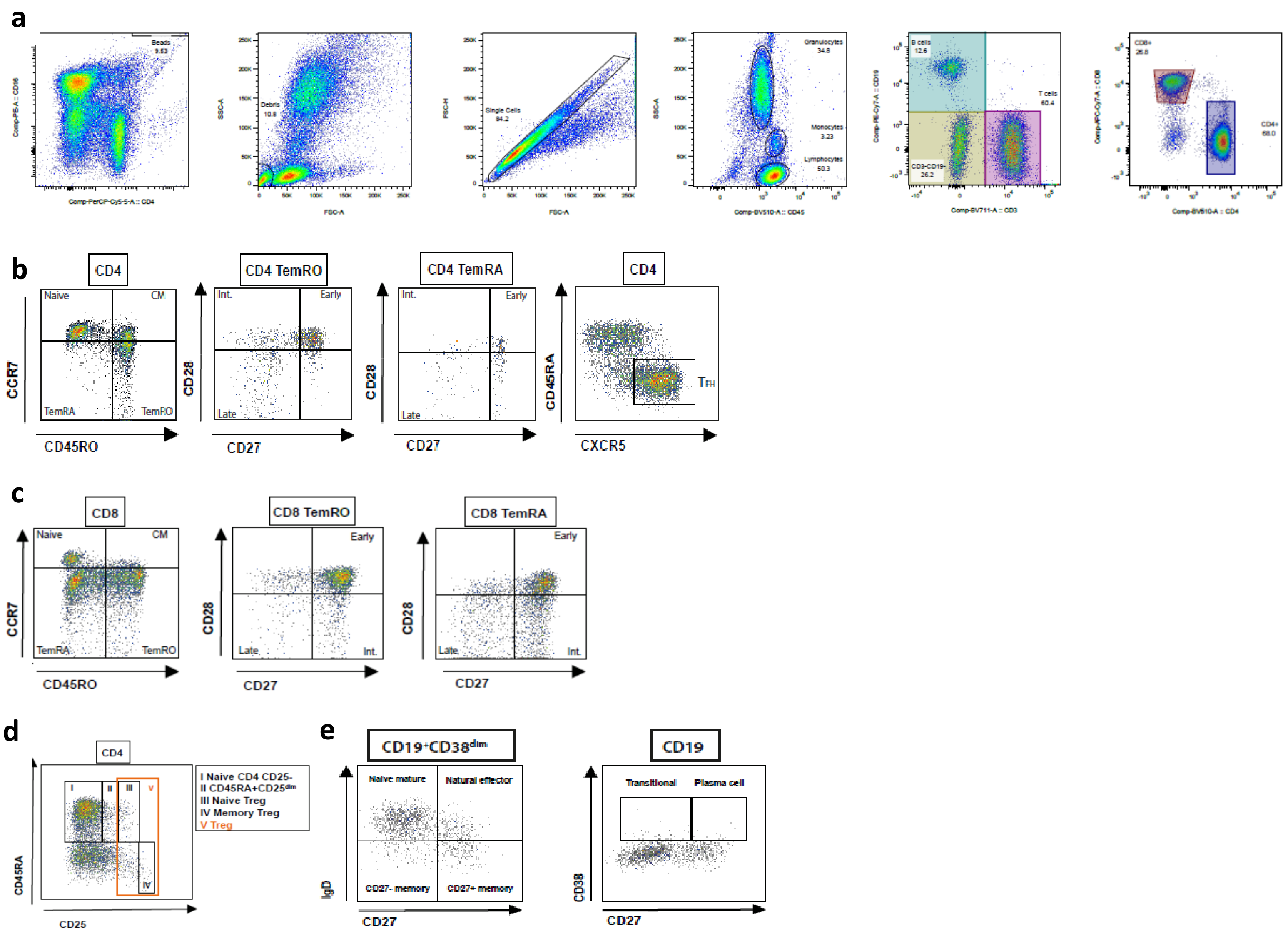

Supplementary Figure 4. Gating strategies

Example gating for the different lymphocyte subsets (**a**), CD4 T-cells subsets (**b**), CD8 T-cell subsets (**c**), Treg subsets (**d**), and B-cell subsets (**e**).
